# Our initial experience of monitoring the autoregulation of cerebral blood flow during cardiopulmonary bypass

**DOI:** 10.1051/ject/2023032

**Published:** 2023-12-15

**Authors:** Leon Andersen, Micael Appelblad, Urban Wiklund, Nina Sundström, Staffan Svenmarker

**Affiliations:** 1 Heart Centre, Department of Public Health and Clinical Medicine, Umeå University 901 87 Umeå Sweden; 2 Department of Radiation Sciences, Radiation Physics, Biomedical Engineering, Umeå University 901 87 Umeå Sweden

**Keywords:** Cardiopulmonary bypass, Monitoring, Cerebral blood flow, Autoregulation, Near-infrared spectroscopy

## Abstract

*Background*: Cerebral blood flow (CBF) is believed to be relatively constant within an upper and lower blood pressure limit. Different methods are available to monitor CBF autoregulation during surgery. This study aims to critically analyze the application of the cerebral oxygenation index (COx), one of the commonly used techniques, using a reference to data from a series of clinical registrations. *Method*: CBF was monitored using near-infrared spectroscopy, while cerebral blood pressure was estimated by recordings obtained from either the radial or femoral artery in 10 patients undergoing cardiopulmonary bypass. The association between CBF and blood pressure was calculated as a moving continuous correlation coefficient. A COx index > 0.4 was regarded as a sign of abnormal cerebral autoregulation (CA). Recordings were examined to discuss reliability measures and clinical feasibility of the measurements, followed by interpretation of individual results, identification of possible pitfalls, and suggestions of alternative methods. *Results and Conclusion*: Monitoring of CA during cardiopulmonary bypass is intriguing and complex. A series of challenges and limitations should be considered before introducing this method into clinical practice.

## Introduction

Cerebral complications after cardiac surgery have a multifaceted background, often with serious consequences for the patient [1]. One risk factor is disturbances of the cerebral circulation during surgery, which may provoke the development of both stroke and delirium [1–3]. Cerebral blood flow (CBF) should under normal conditions be relatively insensitive to variations of the systemic mean arterial pressure (MAP), within a safe upper and lower blood pressure limit [3–5]. The theory of vasomotor control in relation to blood pressure was first demonstrated by Bayliss in 1902 [6] and later manifested by Lassen in 1959 [5]. This principle remains the dominating theory of CBF autoregulation to this day [7, 8].

The brain represents about 2–3% of the total body mass. Despite its relatively small weight it still receives approximately 15% of the systemic blood flow. This disproportionally high CBF is explained by a high cerebral metabolically activity requiring about 20% of available circulating oxygen [9]. As cerebral tissue stores limited energy explains why normal cerebral function depends on a continuous supply of oxygen. How CBF is modulated to meet fluctuating cerebral oxygen demands was demonstrated by Jones and colleagues in 1981 [10]. Normal CBF in conscious humans is 50 mL/100 g/min [9]. A decrease of 50% causes neuronal damage and further reduction can lead to unconsciousness and permanent brain damage [9, 11].

Clinically suitable methods to monitor cerebral autoregulation (CA) emerged during the mid-1990s [12]. The use of near-infrared spectroscopy (NIRS) has later been suggested as a non-invasive method to estimate the CBF [13, 14]. The association between CBF and MAP is assessed by analyzing the correlation between slow waves in NIRS with those in MAP recordings. This association is referred to in the literature as the COx index. The hypothesis is that an increase in COx is indicative of disturbed CA. This technique to monitor CA in real-time recordings has been applied in many studies [3, 4, 13, 15–18] and recently suggested as a reliable method to assess CA [19].

The present study aims to critically analyze and discuss the reliability and validity of the COx index as a measure of CA, where the properties of the COx index were illustrated using data from a series of patients undergoing cardiopulmonary bypass (CPB).

## Material and methods

### Patients

This study was based on data from patients admitted for cardiac surgery at the Heart Centre of Umeå University Hospital between August 2019 and March 2020. The cohort comprised a subset of 22 patients selected at the discretion of the anesthesiologist as suitable for online monitoring of the regional cerebral oxygen saturation. Patients (*n* = 12) requiring deep hypothermic circulatory arrest were however excluded. The remaining cohort (*n* = 10) formed the final dataset. Descriptive characteristics are listed in Table 1.

**Table 1:** Patient characteristics (*n* = 10).

Characteristics	
Age (years)	65 (34)
Male gender (*n*)	10
Body surface area (m^2^)	1.9 (0.4)
Hypertension (*n*)	1
Diabetes mellitus (*n*)	3
Current or prior smoker (*n*)	2
Prior carotid artery disease (*n*)	0
Prior stroke (*n*)	1
Prior transient ischemic attack (*n*)	1
Type of surgery	
Coronary artery bypass grafting (*n*)	2
Composite aortic grafting (*n*)	5
Aortic valve replacement (*n*)	3
Laboratory values	
Haemoglobin (g/L)	147 (18)
Creatinine (μmol/L)	84 (24)
Pre CPB-haematocrit (%)	43.0 (5.8)
Nadir CPB-haematocrit (%)	32.5 (4.3)
Intraoperative notations	
Surgery (min)	254 (106)
Cardiopulmonary bypass (min)	155 (64)
Aortic cross clamp (min)	86 (54)
Nadir body temperature (°C)	33.8 (7.2)
COx	0.02 (0.11)
Mean arterial pressure CPB (mmHg)	58 (6.5)
Cerebral oxygen saturation balance (%)	72 (6.0)

Scale variables: Median (IQR). CPB: Cardiopulmonary bypass.

### Ethics

The Regional Ethical Review Board in Umeå approved the study protocol (DNr-2018/436-31). Patient consent was waived.

### Intraoperative indices

CPB was conducted in a standard fashion maintaining MAP > 50 mmHg, mixed venous oxygen saturation > 70%, non-pulsatile roller pump perfusion (Stöckert S5, LivaNova, London, UK), and membrane oxygenation. Normocapnia (5–6 kPa) and normal arterial oxygen tension (15–20 kPa) were aimed for and verified by intermittent blood gas analyses.

Patient monitoring included at least one arterial blood pressure preferably from the radial artery, central venous pressure via the internal jugular vein, 5-lead electrocardiography, peripheral oxygen saturation, and nasal and/or bladder temperature. Induction of anesthesia was performed using propofol and fentanyl combined with rocuronium for skeletal muscle relaxation. Sevoflurane was used to maintain anesthesia throughout the intraoperative period. Blood pressure was controlled by norephedrine or intermittent boluses of phenylephrine.

### Cerebral oximetry

The INVOS 5100C^TM^ (Medtronic Inc, Solna, Sweden) monitor measures the regional cerebral oxygen balance (rSO_2_) in the prefrontal cortex by emitting infrared light at 730 nm and 810 nm captured by photodetector sensors placed on the left and right side of the forehead. The degree of light absorption at the specified frequencies translates to the actual hemoglobin oxygen saturation level in the arterial, capillary, and venous compartments, where the contribution from the venous saturation is approximately 75% [14, 20]. Median normative rSO_2_ in conscious adult cardiac surgical patients has previously been reported at 66% [21]. It has been established that changes in the rSO_2_ level serve as a good estimate of the CBF when using transcranial Doppler assessments (TCA) as a reference [13, 15–17, 22]. MAP and rSO_2_ were sampled at 0.2 Hz.

### Cerebral oxygenation index

The COx index can either be analyzed in the frequency or time domain [23]. In this study, we focused on analysis in the time domain. The COx index was defined by the calculated Pearson correlation coefficient (*r*) using 30 pairs of MAP and rSO_2_ database recordings updated every 5th second (0.2 Hz). Several different thresholds have been suggested in the literature to identify the loss of CA [24]. In this study, a COx of > 0.4 indicated that CBF is controlled by MAP, whereas COx ≤ 0.4 indicates that CA is preserved and not influenced by MAP variations [2–4, 18]. Collected data were manually filtered to exclude erroneous values following flushing of blood pressure catheters or invalid COx calculations, mostly caused by loss of nominator-denominator variance. Left and right rSO_2_ channels were merged using the mean value for analysis.

The correlation coefficient (*r*) estimates the linear relation between two signals. To estimate if a high correlation reflects a “true” co-variation or only is a random or spurious finding, surrogate data analysis can be applied [25]. The analysis is then repeated after replacing one of the signals with a set of synthesized signals, where the generated signals normally have the same frequency components as the replaced signal. The results can then be used to construct confidence intervals that can be used to evaluate the significance of the results from the interpatient registrations.

To test how often high positive or negative COx values occur in data that are not correlated, we applied surrogate data analysis as follows [25]. For each case, we calculated the COx index based on rSO_2_ and MAP from the same subjects, but for each subject, we also created nine artificial COx values after replacing MAP with data from the other nine subjects. The variation over time in the COx index was then calculated for each combination of rSO_2_ and MAP, where the length of the signals was set to the shortest signal length. Finally, the percentage of the total time when COx was above 0.40 or below −0.40 was calculated for each set of signals, and 90% confidence intervals were constructed based on the 90 artificial COx values, as they represented truly uncorrelated signal pairs. Thus, if the total time with COx > 0.40 is higher than the upper limit of the CI, this would be more frequent than expected if the signals were unrelated, which in turn could indicate a loss of autoregulation.

### Statistical analyses

Calculation of central tendencies, correlations, and production of graphs was performed in Microsoft^Ⓡ^ 365 Excel for Mac (Microsoft Corp, Redmond, WA), SPSS statistical software version 27 (IBM Corp, Armonk, NY), and Matlab R2022a (Mathworks Inc, Natick, MA). Uses of the standard deviation and the median are annotated in the text. Correlations were analyzed using Pearson’s correlation coefficient (*r*).

## Results and discussion

### Understanding the COx index

**Figure 1:**
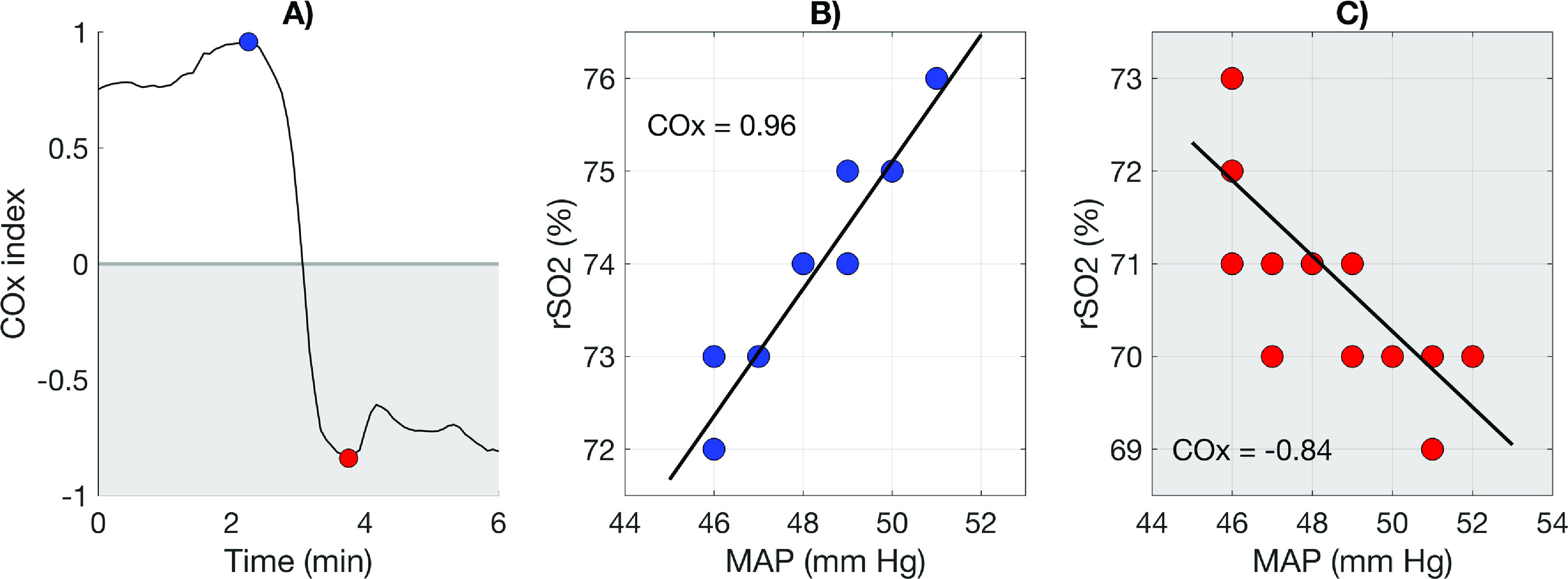
(A) A part of the Cox curve in one of the recordings, with a spread from −0.84 to 0.96. (B) Data representing the blue dot with most positive Cox index. (C) Data representing the red dot with most negative COx index, depending on the independent relation between rSO_2_ and the systemic MAP. Shaded area represents negative Cox index. Unshaded area represents positive COx index. Lines show corresponding linear regression line.

A typical COx curve configuration in the time domain representing the dynamic association [26] between slow wave fluctuations of the estimated CBF and MAP is shown in Figure 1A. Since the COx index is expressed as a correlation coefficient, the range can vary between (−1) and (1). In this illustration, COx varies from (+0.96) to (−0.84), with a sign change occurring after 2 min. A positive COx index appears, when MAP and rSO_2_ are moving in the same direction, i.e.*,* both increase or decrease simultaneously, as indicated in Figure 1B. When CBF dependent on the MAP it implies loss of CA; typically targeted by a COx index > 0.40, while a COx index approaching 0 indicates preserved CA [18]. The paradox of a negative COx index occurs when CBF and MAP move in opposite directions; MAP increases, while CBF decreases or vice versa (Figure 1C). Both these situations are difficult to explain from a physiological standpoint, especially in the case where there is a decrease in MAP and an increase in CBF. This would imply a disproportionable cerebral vascular dilatation to compensate for a cyclic drop in MAP.

The curve formations representing MAP and CBF appear at individual frequencies, which synchronize only occasionally, thus analysis in the time domain infers shifting of these phases [27]. The curve representing CBF is typically delayed in relation to MAP. The effect of phase shifts will inevitably produce significant COx index variations, which may explain why the COx index occasionally turns negative.

**Figure 2:**
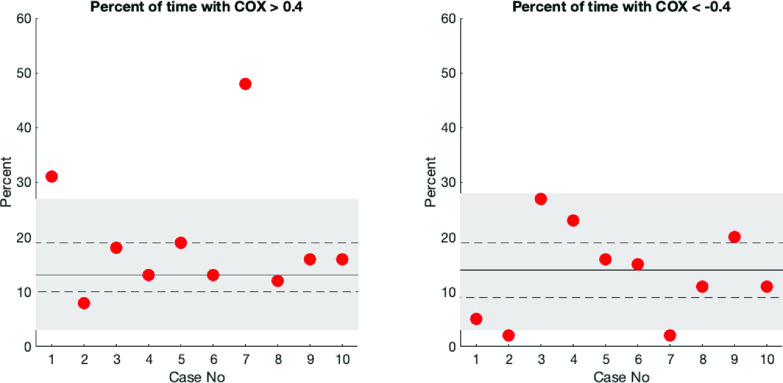
The percent of time with COx > 0.40 or COx < −0.40 in each subject (red dots) related to results based on a unique mixture of 90 uncorrelated signals including all subjects’ individual rSO_2_ signals and a combination of MAP signals from the remaining cases. The 5–95% interval is indicated by the shaded area and the median and quartiles with solid and dashed lines. The red dots represent the results based on the recorded signals in each subject. Two subjects presented more frequent COx > 0.40 than expected by the 95% interval for uncorrelated signals.

The percentage of the total time, when COx was above 0.40 or below −0.40 in individual registrations is presented in Figure 2. The figure also shows the confidence intervals for the corresponding percentages based on the variation in the COx index for the surrogate data set, i.e., the 90 truly unrelated MAP and rSO_2_ signals. The results showed that 8/10 patients were located within the estimated confidence intervals, where the upper limit for the percentage of time with COx > 0.40 was 27%. Interestingly, two patients presented with high COx values in 30–50% of their total recording, which is a more frequent occurrence than expected if their MAP and rSO_2_ signals had been unrelated.

If even short periods with high COx in the recordings from patients is a sign of e.g., an abnormal CA, a phase shift between the signals, or only occurring randomly due to noise warrants further investigation. More studies are also required to evaluate if the surrogate data analysis method is a valid method to identify patients with signs of disturbed CA.

An alternative possible solution would be to combine or replace COx index analysis with either coherence [27] or transfer function analyses [28].

### The COx index from the observer’s eye

**Figure 3:**
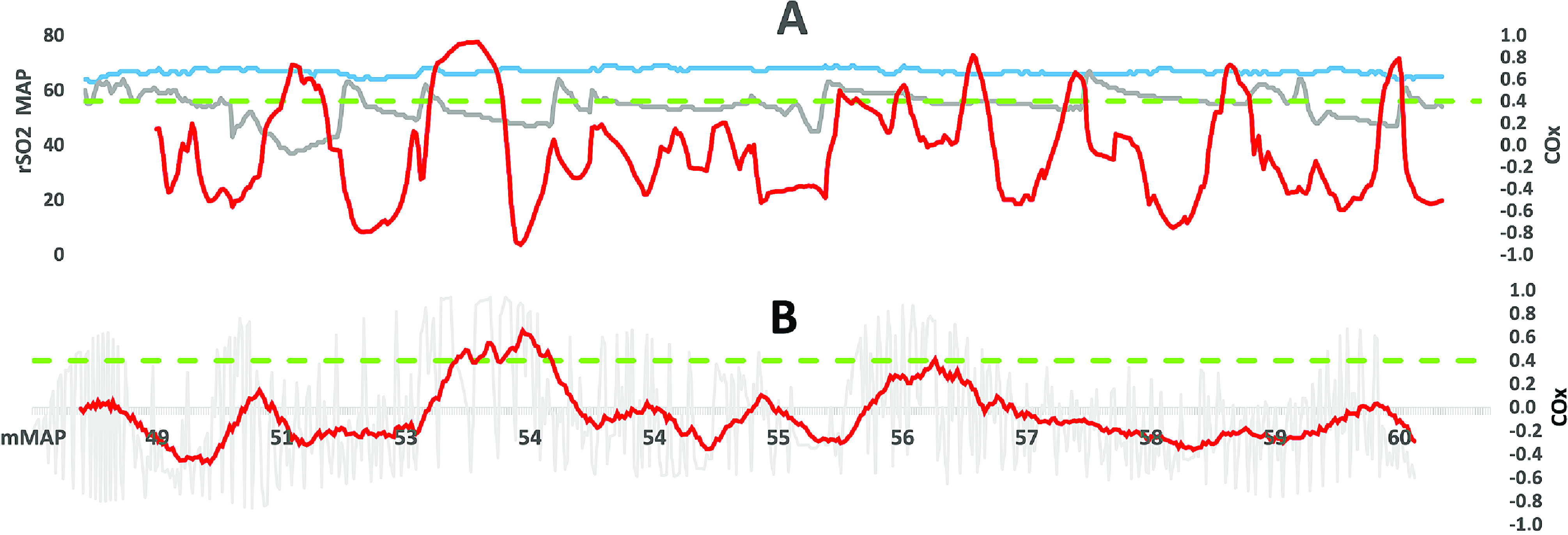
Case example: (A) MAP (grey curve), rSO_2_ (blue curve) and the COx index (red curve) from initiation of cardiopulmonary bypass until weaning (49 min). (B) The MAP sorted from low to high based on the mean value over 2.5-minute intervals (grey curve). The red curve represents the corresponding trended average COx value including 20 registrations. Reference line COx = 0.4 (green dotted line).

The observer’s real-time vision of the information presented in the clinical setting would typically include COx, MAP, and rSO_2_ values shown in Figure 3A. In this example, the duration of CPB is 49 min, during which MAP has ranged from 37 to 67 mmHg, rSO_2_ 63% to 69%, and COx −0.86 to 0.94, respectively. Of note is that both MAP and rSO_2_ remain within acceptable physiological limits, despite a significant COx variation, with loss of CA occurring on eight occasions as defined by the COx > 0.4 criterion. In each case, CA is short-lived and mainly associated with minor changes in MAP, probably due to pharmacological interventions or changes in systemic blood flow or/and vascular resistance. The hypothetical question that remains to be answered is, can these events of abnormal CA be circumvented if the COx index is monitored in real-time? COx values beyond 0.4 in Figure 3A appear during periods of hypotension, followed by a simultaneous elevation of MAP and rSO_2._ The interrogation of our data cannot isolate its cause, however most likely due to a vasoconstrictor intended to normalize the systemic blood pressure.

Adequate interventions related to COx index variations require several considerations. The calculated COx index is not truly instant, it is based on historical data (30 registrations collected over 2.5-minute periods). Before a reliable trend can be visualized, a considerably longer period would be required. It makes instant therapeutic interventions difficult to accomplish, which limits the method’s applicability.

Clinically useful information derived from this method could be to display the COx index in reference to MAP (Figure 3B). This will once again be historical data, however, still possible to gradually build up during a CPB procedure. The rationale behind this would be to identify blood pressure limits (“a safe window”), within which the CA is preserved. Techniques are now available where the actual correlation between MAP and rSO_2_ (COx) is presented online [19, 29, 30].

### Characteristics of variables forming the COx index

**Figure 4:**
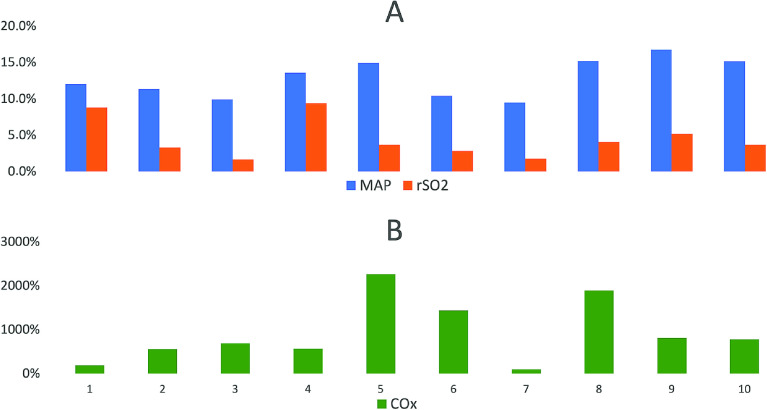
A summary of the variance for MAP, rSO_2_ (A) and the COx index (B) defined by the interpatient (1–10) individual ratio between the mean value and the standard deviation expressed in percent.

Figure 4 shows differences in variability of the parameters used for the computation of the COx index. The variability is defined as the relationship between the standard deviation [*SD*] and the mean value (*MV*) ($\frac{\mathrm{SD}\times 100}{\mathrm{MV}}$) expressed as a percentage (%) of all recordings. The variability of MAP (13%) overweighed the variability of rSO_2_ (4%) by more than 300% based on the interpatient overall median values (Figure 4A). The difference is of note, as it means that the resulting COx index is more sensitive to changes in MAP than rSO_2_. This is also evident from the illustration in Figure 3. Figure 4B further illustrates how MAP influences the COx index with the recorded interpatient variability ranging from 95% to 2264%, with a median value of 779%.

### Posthoc analysis of the COx index

**Figure 5:**
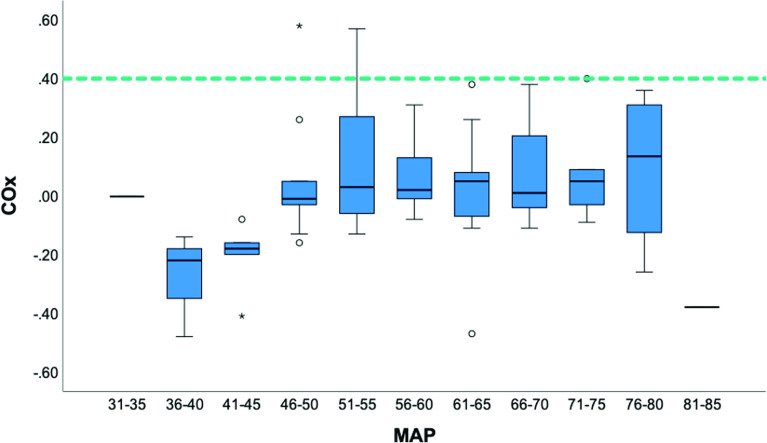
Boxplots showing COx sorted in bins of MAP including all patients (*n* = 10). COx = 0.4 (green dotted line). The boxplot illustrates the interquartile range (IQR) and the median value (horizontal line). Whiskers represent minimum and maximum values. ο = 3^rd^ quartile + 1.5*IQR and 1^st^ quartile −1.5*IQR. * = 3^rd^ quartile + 3.0*IQR and 1^st^ quartile −3.0*IQR.

The COx index presented in publications is generally calculated posthoc, thus, it does not reflect real-time observations, and this is one of the main objections to using the method to monitor [19, 29]. By plotting the COx index in consecutive bins of MAP, the focus is to identify the lower and upper limits of MAP, i.e.*,* within what range of MAP CA is intact. Such a limit was identified in 1 out of 10 patients in our series, with a lower blood pressure limit of 55 mmHg for COx > 0.4 as reference (Figure 5). An accompanied higher blood pressure limit was, however, not detected in any subject, why a “safe window” of MAP could not be established. In this patient, loss of CA lasted for 11 min, a value representing a summary of 15 independent short time intervals, which is important to bear in mind. In fact, it would seem reasonable to detect thresholds of abnormal CA in all patients, especially for lower blood pressure limits, since episodes of hypotension are frequent during a CPB procedure. The MAP ranged between 46 mmHg and 63 mmHg in our series but, individual MAP recordings as low as 23 mmHg were observed, contrasted by relative hypertensive events up to 95 mmHg. Why this full range of MAP recordings was unable to identify blood pressure limits for the maintenance of CA may raise the questions about method’s validity.

**Figure 6:**
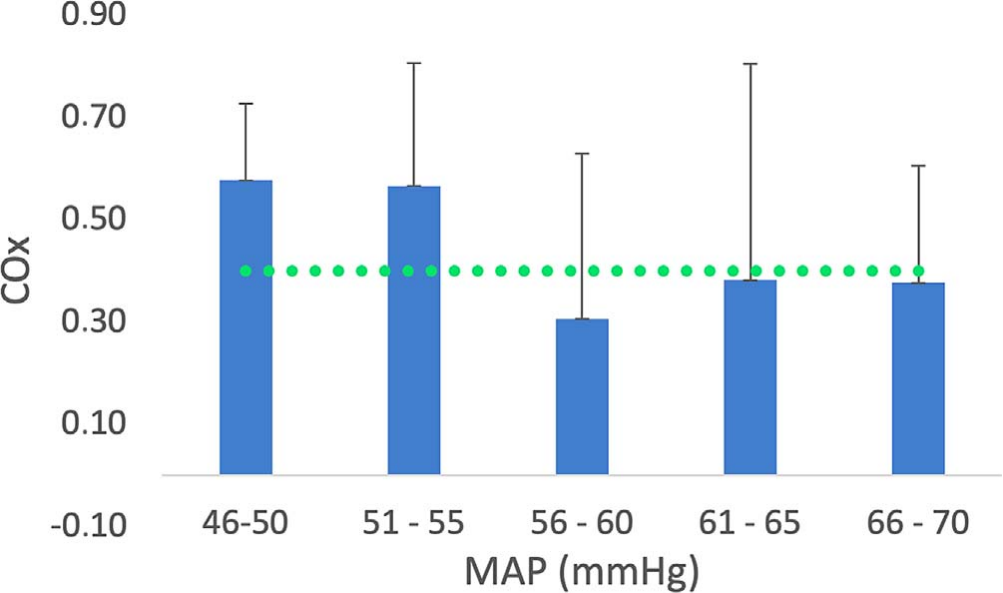
Illustration of COx index recordings in patient plotted in bins of MAP. Lower MAP limit for preserved CA identified at 55 mmHg. Limit for disturbed autoregulation: COx > 0.4 (green dotted line). Error bars show the standard deviation.

An overview of the COx index relative bins of MAP including all patients is shown in Figure 5. This indicates that CA was maintained over the entire span of blood pressure. It should however be underlined that the presentation disguises patients’ individual COx values indicating loss of CA exemplified in Figure 6. Nevertheless, it gives a fair illustration of the COx index distribution. MAP ranged between 30 mmHg and 90 mmHg in our patients, still, the COx method signaled sustained CA. This finding alone raises questions about the method’s sensibility. The other plausible answer to this puzzling result would be to question the role of MAP in relation to CA during CPB [31, 32].

### Setting the COx index threshold

On the critical evaluation of the threshold for the COx index, which is typically set to >0.4 [18, 33], it should be noted that a correlation coefficient (*r*) at this level indicates a weak association, where the degree of explanation (*r*^2^) equals just 16%. From a statistical viewpoint, there is room for a significant degree of uncertainty. Increasing the COx threshold above the 0.4 level would probably increase the sensitivity, but at the same time significantly decrease the success rate, when loss of CA is detected. Where the balance between the magnitude of COx and the success rate is optimal is yet to be defined. The influence of a constantly high rSO_2_ is of interest. It may explain, why only a few patients demonstrated abnormal CA.

The COx threshold >0.4 to define impaired CA has become more of a gold standard [24]. However, this threshold was obtained from animal experiments performed in the naive piglet brain [16, 18]. In these experiments, MAP was gradually decreased by inflating a balloon catheter placed in the inferior vena cava as a method to identify the lower blood pressure limit of CA. The balloon placed in the inferior cava obstructed the venous return and lowered the filling pressure of both the right and left ventricles. The observed decrease in MAP was, therefore, most likely caused by a simultaneous decrease in cardiac output [34, 35]. Cardiac output was unfortunately not reported during these experiments so this cannot be confirmed. It is difficult to isolate the circulatory effects of MAP manipulations even in the setting of an animal experiment, without interfering with other complex regulatory mechanisms and in this case specifically related to the influence on the CBF.

### Autoregulation of cerebral blood flow and cardiopulmonary bypass

The systemic blood flow is established by the pressure wave generated by cardiac muscle contractions. The pressure build-up within the left and right ventricle during systole is continuous until it overtakes the backpressure on the other side of the opening aortic and pulmonary valve. The ejected stroke volume will elevate the systemic and pulmonary blood pressure at two completely different levels directly related to the vascular resistance in the respective circulatory beds. Of note is that the cardiac output will still be the same within both vascular beds, despite these key differences [36]. The relative difference in blood pressure between the systemic and pulmonary circulation is five-fold [37]. What this tells us is that the blood pressure *per se* does not say anything about its relation to the actual blood flow unless vascular resistance is known.

This reasoning can be applied in the process of CPB. The main objective in this context is to establish and maintain adequate systemic blood flow traditionally based on a fixed index related to the patient’s body surface area [38] or more recently to balance the patient’s global oxygen demand [39, 40]. Blood pressure is still important but is adjusted secondary to the systemic blood flow demand. The approach is in total contrast to overall general clinical practice where blood pressure is the focus and represents the gold standard for the evaluation of patient health and circulatory status [41]. Blood pressure measurements are easily accomplished, combined with a strong historical impact, which also explains its central role in clinical medicine.

**Figure 7:**
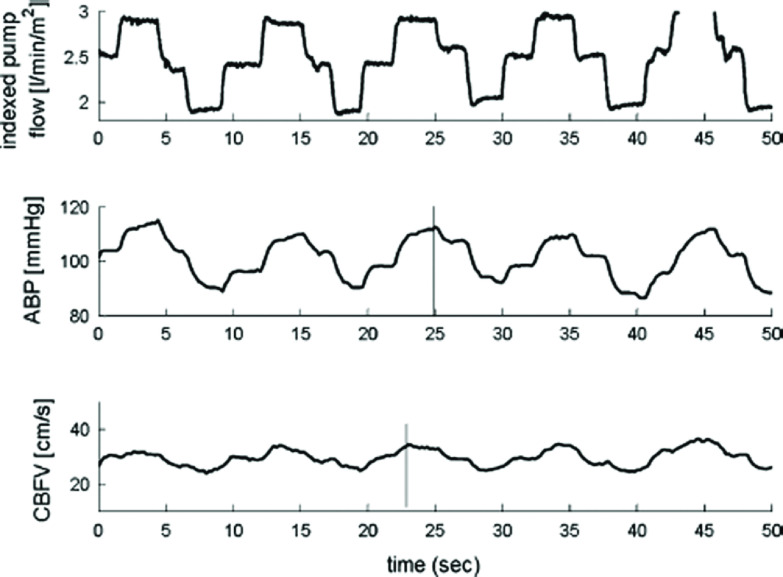
Effect of cyclic pump flow changes (upper panel) on arterial blood pressure (ABP, middle panel), and cerebral blood flow velocity (CBFV) in the middle cerebral arteries (lower panel) at PaCO_2_ of 40 mmHg (5.3 kPa) in a single patient undergoing cardiopulmonary bypass. Reprinted with permission from the owner Springer Nature: Ševerdija EE, Gommer ED, Weerwind PW, Reulen JP, Mess WH, Maessen JG (2015) Assessment of dynamic cerebral autoregulation and cerebral carbon dioxide reactivity during normothermic cardiopulmonary bypass. Med Biol Eng Comput 53(3), 195–203.

The systemic blood flow delivered from the heart-lung machine will be distributed in a hierarchical manner to all organs and vascular beds [42]. With respect to cerebral circulation, what would be most important: “flow or pressure”? Anyone with clinical experience from CPB will know that a momentous change in the systemic blood flow will respond with a reciprocal momentous change in systemic blood pressure. Therefore, it is difficult to isolate one component from the other; it merely confirms that these components are closely linked. Is this observation of scientific relevance? Probably yes, at least with reference to dynamic pressure variations measured in the time domain. Ševerdija et al. showed how variations of the systemic blood flow at 0.1 Hz during CPB covariated with both the CBF and the systemic blood pressure verified by the graphical representation in Figure 7 [43].

Much of our understanding regarding how CBF control is from the seminal publication by Lassen in 1959 [5]. Extrapolation of cross-sectional comparisons from 11 groups including (a total of 376 subjects) across 7 publications was presented in a graph showing the relation between systemic blood pressure and CBF. Only two data points formed the threshold for a lower blood pressure limit and none for the upper limit. The remaining were aggregated forming a straight line. Despite these formal limitations regarding the research methodology, blood pressure is still regarded as one of the essential components for autoregulation of the CBF. An updated interpretation of Lassen’s autoregulation curve suggests it only applies to more gradual amendments of the blood pressure, which will have little or no effect on the CBF. Examples are the treatment of hypertension or the downregulation of the blood pressure occurring during nocturnal sleep [11].

Even if systemic blood flow and blood pressure are essential in relation to CBF control, other equally essential regulatory mechanisms are also involved, such as neurovascular coupling [44], cerebrovascular carbon dioxide tension, and oxygen reactivity [45]. This emphasizes the fact that CA should not be evaluated from the perspective of one of these regulatory mechanisms alone as CA refers to a complex interaction between all involved [11].

### Clinical outcome related to the COx index

The amount of evidence showing an absolute association between results from monitoring the COx index and clinical outcomes is limited. Brown and colleagues assessed CA by means of transcranial Doppler in a group of cardiac surgical patients requiring the use of CPB [46]. A total of 20.6% of the patients were observed with episodes of CA dysfunction. The lower mean arterial blood pressure limit of CA was targeted prior to CPB using mean velocity index (Mx) > 0.4 – equivalent to the COx threshold. The computed blood pressure target ranged 35.0–97.5 mmHg, which may seem extraordinary. Patients randomized to maintain MAP above the lower designated blood pressure limit of CA during CPB were less likely to develop postoperative delirium. Whether this was caused by the MAP target or a longer duration of normal CA was unfortunately not discussed. These findings agree with those reported by Chan and Aneman [47]. In this prospective study, COx was registered on the two first following postoperative days. The COx index was significantly higher among patients diagnosed with postoperative delirium on postoperative Day 1: 0.270 ± 0.199 vs. 0.180 ± 0.142 (*P* = 0.044). The magnitude of these COx indices is however very low, which generally would not indicate disturbed CA. Hori and co-workers showed how a blood pressure below the lower limit of preserved CA observed in the intensive care unit was positively correlated with the release of the brain-specific injury biomarker glial fibrillary acidic protein (GFAP) [48]. Of note is that blood pressure targets for the lower limit of preserved CA were calculated during CPB, while outcome measures were performed postoperatively. This is once again an example of an indirect conclusion being drawn from an association between blood pressure and outcome; not how fluctuations of CA per se correlated with the outcome – in this case the release of GFAP. Blood pressure excursions below the lower limit of CA during CPB have also been shown to be associated with major morbidity and intraoperative mortality [49]. Using a composite variable to identify risk factors associated with abnormal CA makes it difficult to verify a causal relationship. In addition, accepting COx ≥ 0.3 as the threshold of abnormal CI involves a significant degree of uncertainty, which would only explain about 10% of the observed correlation between the measured MAP and CBF. Among 450 patients, 83 experienced complications according to the a priori definition. The risk of developing complications was independently associated with the area under the curve for the duration of MAP below the lower limit of preserved CA.

### Closing remarks

Monitoring the CA by measuring the correlation between CBF and MAP is intriguing. The method aims to identify a blood pressure limit at which MAP and CBF become correlated. Numerous publications verify that this is possible and more importantly that the information can be used to protect patient groups against a variety of different adverse complications. The key question is whether this blood pressure limit really is a reliable marker of CA? To answer this question, we need investigations to verify that the blood pressure limit can be used to successfully tailor and maintain CA in real-time, i.e.*,* if MAP drops below the targeted limit, the COx registration should signal, and additionally verify that CA is restored by blood pressure adjustments. To the best of our knowledge, no such verification publication exists. Until then, we believe that the information obtained from the COx index should be thoroughly scrutinized before introduction into clinical practice.

## Data Availability

Data supporting the results from this study are available on reasonable request from the authors.
